# Salvianolic Acid C Protects against Cisplatin-Induced Acute Kidney Injury through Attenuation of Inflammation, Oxidative Stress and Apoptotic Effects and Activation of the CaMKK–AMPK–Sirt1-Associated Signaling Pathway in Mouse Models

**DOI:** 10.3390/antiox10101620

**Published:** 2021-10-15

**Authors:** Liang-Hsuan Chien, Chien-Ta Wu, Jeng-Shyan Deng, Wen-Ping Jiang, Wen-Chin Huang, Guan-Jhong Huang

**Affiliations:** 1 Department of Chinese Pharmaceutical Sciences and Chinese Medicine Resources, College of Chinese Medicine, China Medical University, Taichung 404, Taiwan; u107047001@cmu.edu.tw; 2 Faculty of Pharmacy, College of Pharmacy, Kaohsiung Medical University, Kaohsiung 807, Taiwan; u105830013@kmu.edu.tw; 3 Department of Health and Nutrition Biotechnology, Asia University, Taichung 413, Taiwan; dengjs@asia.edu.tw; 4 Department of Pharmacy, Chia Nan University of Pharmacy and Science, Tainan 717, Taiwan; wpjiang@gm.cnu.edu.tw; 5 Graduate Institute of Biomedical Sciences, School of Medicine, China Medical University, Taichung 404, Taiwan; huangwc@mail.cmu.edu.tw; 6 International Master’s Program of Biomedical Sciences, School of Medicine, China Medical University, Taichung 404, Taiwan

**Keywords:** salvianolic acid C, cisplatin, acute kidney injury, anti-inflammation, oxidative stress, apoptosis, CaMKK–AMPK–Sirt1 pathway

## Abstract

Acute kidney injury (AKI) is a sudden reduction in kidney activity and has a high mortality rate. Salvianolic acid C (SAC), one of the main polyphenolic components of *Salvia miltiorrhiza*, displays significant pharmacologically active effects. An animal model of cisplatin-induced kidney injury was used to study the potential of SAC to improve AKI. First, SAC was administered intraperitoneally in mice for 10 consecutive days, and then cisplatin was administered intraperitoneally on day 7 to establish a nephrotoxicity mouse model. SAC mitigated renal histological changes, blood creatinine (CRE) and blood urea nitrogen (BUN) production and the levels of inflammatory mediators in the cisplatin-induced AKI. Furthermore, malondialdehyde (MDA) levels were reduced and glutathione (GSH) was increased after intraperitoneal injection (i.p.) administration of SAC. In addition, based on Western blot data, SAC reduced the expression of inducible NO synthase (iNOS), cyclooxygenase-2 (COX-2), nuclear factor kappa B (NF-κB) and mitogen-activated protein kinase (MAPK) activation in mouse renal tissues. Finally, SAC diminished the level of TLR-4 expression and enhanced the production of several antioxidative enzymes (superoxidase dismutase (SOD1), glutathione peroxidase (GPx3), catalase, nuclear-factor-erythroid-2-related factor 2 (Nrf2) and heme oxygenase 1 (HO-1)), Sirtuin 1 (Sirt1), p-AMP-activated protein kinase (AMPK) and p-Ca^2+^/calmodulin-dependent protein kinase kinase (CaMKK). In addition, Sirt1 inhibition (EX 527) inverted the effect of SAC against cisplatin-induced nephrotoxicity. Collectively, SAC provides a therapeutic target with promising clinical potential after cisplatin treatment by attenuating oxidative stress and inflammation.

## 1. Introduction

Cisplatin (cis-diamminedichloroplatinum II), a platinum compound, is currently applied singly or in combination with other medicines for treating cancers. However, the use of cisplatin is frequently limited by its adverse consequences, such as myelosuppression, nephrotoxicity and peripheral neuropathy [1]. Cisplatin administration for acute kidney injury (AKI) leads to serious side effects and there is a higher risk of death [2]. In clinical practice, the main causes of AKI are infections, nephrotoxic drugs, diabetes, hypertension, sepsis and cardiovascular disease. Currently, chemotherapy combined with cisplatin is the most common treatment for cancer therapy. However, the combination of cisplatin and cancer treatment might exacerbate kidney damage as AKI is also a general side effect for most patients receiving cancer treatment. Thus, it is necessary to prepare new and effective strategies to prevent and treat AKI.

The molecular basis of cisplatin-induced AKI is not fully clear, and mounting evidence points to oxidative stress and inflammation leading to AKI. Cisplatin has been found to produce a lot of free radicals in renal cells, which destroy cellular structures and cellular components, so antioxidant defense is one of the key signs of cisplatin-induced nephrotoxicity [3].

TLRs are expressed in renal tissue and in infiltrating inflammatory cells. In the kidney, tubular epithelial cells and mesangial cells express TLR-1, TLR-2, TLR-3, TLR-4 and TLR-6 and podocytes express TLR-1, TLR-2, TLR-3, TLR-4, TLR-5, TLR-6 and TLR-10 [4]. Cisplatin-induced renal toxicity is mediated in part through toll-like receptor 4 [5]. The signal transduction initiated by TLRs activates effector cells of the innate immune system via several kinases and NF-κB and generates pro-inflammatory cytokines [6].

Aggravation of reactive oxygen species (ROS) causes AKI through ROS-induced atypical signaling pathways, inflammatory infiltration, cell disorder and renal cell mortality [7,8]. Cytokines and ROS play an important role in cisplatin-associated AKI. AKI has been reported to be related with pro-inflammatory cytokines, including interleukin-1β (IL-1β), interleukin-6 (IL-6) and tumor necrosis factor-α (TNF-α) [9,10]. These oxidative stress and inflammatory mediators can damage renal tubule cells and renal tissues. Thus, attenuation of inflammatory reaction and oxidative stress could be a potential approach to curing cisplatin-associated AKI. In addition, ROS enhances inflammation through nuclear factor (NF)-κB, nuclear-factor-erythroid-2-related factor 2 (Nrf2), other MAPK kinases and redox-sensitive transcription factors [11,12]. Therefore, treatment aimed at concurrently restraining NF-κB, MAPKs and the Nrf2–HO-1 axis could serve as a potential therapy for AKI.

Adenosine 5′-monophosphate-activated protein kinase (AMPK) is triggered by a reduced energy state of the cell, such as an increased AMP/ATP ratio. Silent information regulator T1 (Sirt1), a NAD+-dependent histone deacetylase, is related to cell metabolism and considered a metabolic sensor. In terms of biological functions, Sirt1 has been demonstrated to control apoptosis, cell proliferation, DNA repair, autophagy and tumorigenesis [13,14,15]. Calcium/calmodulin-dependent protein kinase (CaMKK) is a key CaM kinase driven by increased intracellular calcium and plays a major part in the calcium-mediated regulation of inflammation in innate immune cells [16]. Recent studies have revealed that AMPK also effectively inhibits NF-𝜅B signaling and is related to the inflammatory response of macrophages [17]. In metabolism, the major downstream regulator of AMPK is Sirt1 [18]. Sirt1 is also needed for AMPK-mediated inflammation inhibition [19], and previous research indicates that the CaMKK–AMPK–Sirt1 axis plays a significant role in disease therapy [16,17,18,19].

*Salvia miltiorrhiza*, an important herb in traditional Chinese medicine, has been extensively used in China for thousands of years to treat cardiovascular and cerebrovascular diseases. Salvianolic acid C (SAC) is a water-soluble active component isolated from *S. miltiorrhiza*, and its polyphenolic structure shows significant antioxidant capacity, important for treating inflammation and cardiovascular diseases [20]. SAC inhibits the activity of HMG-CoA reductase, which is a major target for the treatment of hypercholesterolemia [21]. Moreover, SAC inhibits NF-κB activity in endothelial cells and improves the process of aortic aneurysm [22]. Our previous paper showed that SAC reduces inflammation, oxidative stress and caspase-mediated apoptosis by inactivating Keap1/Nrf2/HO-1 signaling in acute liver injury [23,24]. Thus, in the present study, we evaluated the antioxidant and anti-inflammatory effects of SAC in cisplatin-associated nephrotoxicity mouse models. SAC might be a good adjuvant therapy for cancer once how to prevent AKI has been determined.

## 2. Materials and Methods

### 2.1. Reagents

**Figure 1:**
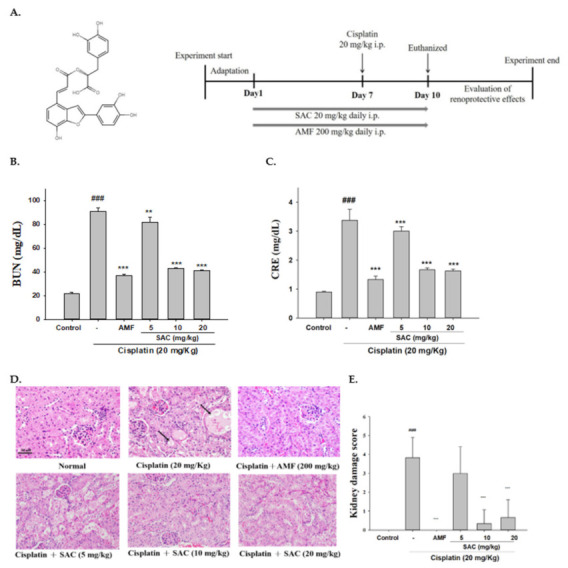
Structure of SAC and experiment design (**A**) and protective effects of SAC on cisplatin-induced AKI in mice. SAC given to mice at a daily intraperitoneal (i.p.) dose of 5, 10 and 20 mg/kg over 10 days; SAC was administered first on day 7, followed 1 h later by cisplatin, and the mice were sacrificed on day 11. Blood BUN levels (**B**) and CRE levels (**C**). Kidneys stained with H&E (**D**) and the kidney injury scores (**E**). Each group’s kidneys were provided for histological evaluation. After H&E staining, representative histological sections were magnified (400×) and photographed. The values are reported as the mean ± SEM (*n* = 6). ^###^ *p* < 0.001 compared with the control group. ** *p* < 0.01 and *** *p* < 0.001 compared with the cisplatin-only group. Arrows show tubular cell necrosis; scale bar = 50 μm.

SAC (purity 98.6%) (Figure 1A) was obtained from Chem Faces Pharmaceutical Company (Wuhan, China). Cisplatin, amifostine (AMF), EX-527 and other solvents and reagents were purchased from Sigma-Aldrich (St. Louis, MO, USA). BUN and CRE assay kits were supplied by HUMAN Diagnostics Worldwide (Wiesbaden, Germany). ELISA Max TM Set Deluxe kits, used to test IL-1β, IL-6 and TNF-α in mice, were obtained from BioLegend Inc. (San Diego, CA, USA). For Western blotting, primary antibodies that contrapose COX-2, p-JNK, catalase, SOD1, Sirt1, AMPK, GPx3 and TLR-4 were bought from GeneTex (San Antonio, TX, USA). Antibodies against JNK, p-ERK, ERK, p-p38, p-CaMKK, p-AMPK and p-IκB-α were bought from Cell Signaling Technology (Beverly, MA, USA). Antibodies that contrapose iNOS, NF-κB, IκBα, HO-1, Nrf2, p38 and β-actin were bought from Abcam (Cambridge, UK, USA). β-actin is used as an endogenous control protein.

### 2.2. Animals

Mice (male ICR, age 6–8 weeks, weight 20–25 g) were provided by BioLASCO Taiwan Co., Ltd. Before the experiment, the mice were kept in a 12 h/12 h light/dark environment at 23 ± 2 °C and relative humidity 50–60% for 1 week. The Animal Care Committee of China Medical University permitted the process for the entire experiment, and the IACUC approval number is 2018–280.

### 2.3. Research Design

The mice were divided into six groups (*n* = 6) at random: control, cisplatin (20 mg/kg body weight, i.p.), amifostine (AMF; 200 mg/kg, i.p.) + cisplatin, SAC (5 mg/kg, i.p.) + cisplatin, SAC (10 mg/kg) + cisplatin and SAC (20 mg/kg) + cisplatin. The groups were adapted to the environment for 1 week. The mice were i.p.-treated with SAC for 10 sequential days. The mice in the control group were given saline. On day 7, to induce AKI in the mice, the mice in the cisplatin group and the SAC-treated group were treated to a single intraperitoneal (i.p.) injection of cisplatin (20 mg/kg body weight) 30 min after treatment with SAC. On day 10, after cisplatin injection, whole blood was collected and the mice sacrificed. The blood was centrifuged at 4 °C (2000 g, 15 min) to retrieve the serum and stored the serum at −20 °C. The kidneys were immediately collected for subsequent assays. During this study, clinical symptoms were checked twice daily. During this period, the weight was measured weekly and the average was calculated.

To evaluate the role of EX-527 in regulating cisplatin-induced AKI, the mice were randomly divided into five groups (*n* = 6 per group): control, cisplatin (20 mg/kg), cisplatin + EX-527 (10 mg/kg), cisplatin (20 mg/kg) + SAC (20 mg/kg) and cisplatin (20 mg/kg) + SAC (20 mg/kg) + EX-527 (10 mg/kg). The mice were administered SAC (20 mg/kg) for 10 sequential days. The mice in the control group were given saline. On day 7, in order to induce AKI in the mice, the mice of the cisplatin group and the SAC-treated group were treated to a single i.p. injection of cisplatin (20 mg/kg) 30 min after treatment with SAC. EX-527 (10 mg/kg) was given intraperitoneally to the animals of the intervention groups 1 h prior to cisplatin administration. All mice were sacrificed 72 h after cisplatin injection.

### 2.4. Assess Kidney/Body Mass Index

The mice body weights were calculated before euthanasia; then, the renal systems were operatively separated and weighed, with kidney/body mass index being measured as follows: kidney weight (g)/body weight (g)

### 2.5. Renal Function Tests

In accordance with the maker’s instructions, serum BUN and CRE were measured using a chemical analyzer (Roche Diagnostics, Cobas Mira Plus, Germany).

### 2.6. Histopathological Analysis

Renal tissue was formalin-fixed, embedded in paraffin blocks, cut into 5 μm thick sections and treated with H&E and then photos taken by light microscopy (Nikon, ECLIPSE, TS100, Tokyo, Japan). According to the degree of epithelial damage in the renal cortical tubules, it is divided into five grades (normal kidney, <25% injury, 25–50% injury, 50–75% injury and >75% injury) and scored from 0 to 4 [25].

### 2.7. TUNEL Staining

Paraffin slices from each group were stained with terminal deoxynucleotidyl-transferase-mediated dUTP nick end labeling (TUNEL) staining. TUNEL staining was performed using TUNEL apoptosis detection kits (Roche Molecular Biochemicals, Indianapolis, IN, USA), according to the manufacturer’s instructions, and photographed with a light microscope (Leica DM750, Solms, Germany).

### 2.8. Lipid Peroxidation Assays

The thiobarbituric acid (TBA) response was detected by measuring malondialdehyde (MDA) levels of renal lipid peroxidation [26]. Briefly, the kidney was homogenized with lysis buffer on ice. To form the MDA–TBA adduct, TBA solution was added to each sample and the mixture incubated at 90 °C for 45 min. Then, each reaction mixture was put into a 96-well plate to measure the absorbance at 532 nm.

### 2.9. Cytokine Assay

In accordance with the maker’s instructions, the serum levels of some pro-inflammatory cytokines were evaluated using an ELISA kit (BioLegend, San Diego, CA, USA).

### 2.10. Nitrite Assay

The Griess reaction colorimetric method was used to determine the nitrite concentration [27]. Concisely, 100 μL of Griess reagent was added to the culture supernatant and the solution mixed and incubated at 540 nm for 10 min to measure the absorbance with a micro-plate reader (Molecular Devices, Orleans Drive, Sunnyvale, CA, USA).

### 2.11. Glutathione Estimation

The DTNB (5,5’-dithiobis (2-nitrobenzoic acid)) assay method can measure the GSH content. We used 10% trichloroacetic acid buffer to homogenize the tissues and then centrifuged it at 1500× *g* and 4 °C for 10 min to obtain the supernatant. For sample pretreatment, 100 μL of the supernatant, 200 μL of 0.3 M phosphate buffer (pH 8.4), 400 μL of double-distilled water and 500 μL of DTNB were mixed beforehand. After the addition of DTNB at 412 nm, optical density (OD) was used as a measure of the control reagent blank. To determine the concentration of GSH, a curve measured with a known quantity was used [28]. The protein content was determined using protein assay kits (Bio-Rad Laboratories, Hemel Hempstead, UK).

### 2.12. Western Blot Analysis

Tissues were homogenized and protease inhibitors used for lysis. A Bio-Rad protein assay kit (BioRad, Hercules, CA, USA) was used to determine the concentration of protein and prepare the sample for Western blot analysis. For electrophoresis, 50 µg of the proteins was added to each lane on the 12% SDS polyacrylamide gel. After that, it was transferred to a PVDF membrane. Appropriate secondary antibody, horseradish peroxidase (HRP) conjugate (Sigma, St. Louis, MO, USA), was selected to combine with the ECL substrate (Amersham International plc., Buckinghamshire, UK) and its signal detected by using Kodak Molecular Imaging Software (Eastman Kodak Company, Rochester, NY, USA).

### 2.13. Statistical Analysis

All the values were expressed as the mean ± standard error of the mean (SEM). Data were analyzed using SPSS software 21.0 (SPSS, Inc., Chicago, IL, USA). The results of Western blot were quantified by Image J (National Institutes of Health, Bethesda, MD, USA). Data under the banks presented as the mean of three results of Western blot. The difference between the two groups was compared by student’s *t* test, and one-way ANOVA was used for the analysis of multiple group data. The *p*-value was divided into three levels, <0.05, <0.01 and <0.001, all of which were considered significant.

## 3. Results

### 3.1. SAC Inhibits Renal Damage and Improves Renal Function in Mice with Cisplatin-Induced Renal Injury

CRE and BUN are active markers of kidney function. As shown in Figure 1B,C SAC (5, 10 and 20 mg/kg) pretreatment inhibited the increase in serum CRE and BUN induced by cisplatin. AMF, a phosphorylated carbitol, has been used in several cancer treatments to raise the quality of life of cancer patients and decrease the adverse effects of anticancer drugs. In this study, we used AMF as a positive control. Collectively, the data show that SAC enhanced the renal activity and kidney structure in mice with cisplatin-induced AKI.

Next, we determined whether SAC improves cisplatin-induced AKI by analyzing the histopathological alterations in kidney tissues. As shown in Figure 1D, the control group had typical tubular and glomerular architectures. The cisplatin group showed severe damage, inflammatory cell infiltration, tubular epithelial damage, focal vacuolar degeneration and necrosis. In contrast, these histological alternations were diminished in the SAC group; i.e., SAC reduced kidney damage in mice (Figure 1D,E). The renal damage scores were also reduced in SAC-pretreated AKI mice compared with mice with cisplatin-induced AKI (Figure 1E). Thus, SAC improves kidney activity and structure in mice with cisplatin-induced AKI.

### 3.2. Changes in the Renal Index of SAC-Protected Mice Treated with Cisplatin

The kidney index is a biomarker of cisplatin-induced renal injury. As shown Table 1, cisplatin-treated mice had lower body weights and an increase in the relative kidney indexes compared to the control mice. However, mice pretreated with SAC showed a significantly higher resistance to cisplatin-induced nephrotoxicity, such as a reduction in the kidney index.

### 3.3. SAC Decreases NO and Pro-Inflammatory Cytokine Serum Levels in Cisplatin-Associated Nephrotoxicity

**Figure 2:**
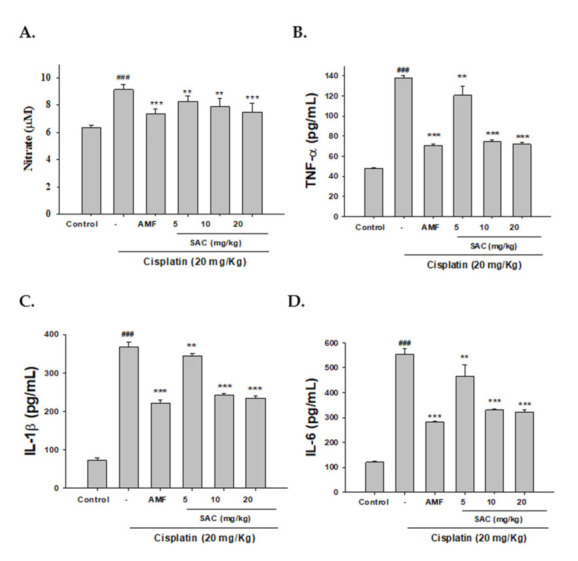
SAC inhibited (**A**) NO, (**B**) TNF-α, (**C**) IL-1β and (**D**) IL-6 serum levels in cisplatin-induced nephrotoxic mice. Griess reaction was used to measure nitrite concentration. ELISA kits were used to measure serum levels of TNF-α, IL-1β and IL-6. The values are reported as the mean ± SEM. (*n* = 6). ^###^ *p* < 0.001 compared with the control group. ** *p* < 0.01 and *** *p* < 0.001 compared with the cisplatin-only group.

As shown in Figure 2A–D, cisplatin-induced nephrotoxic effects appeared as elevated levels of NO, TNF-α, IL-1β and IL-6 compared to the control. Pretreatment with SAC and AMF inhibited NO, TNF-α, IL-1β and IL-6 production after cisplatin treatment. Collectively, the data demonstrate that SAC resulted in a significant decrease in the pro-inflammatory cytokine levels.

### 3.4. SAC Diminishes Oxidative Stress in Cisplatin-Associated Nephrotoxicity

**Figure 3:**
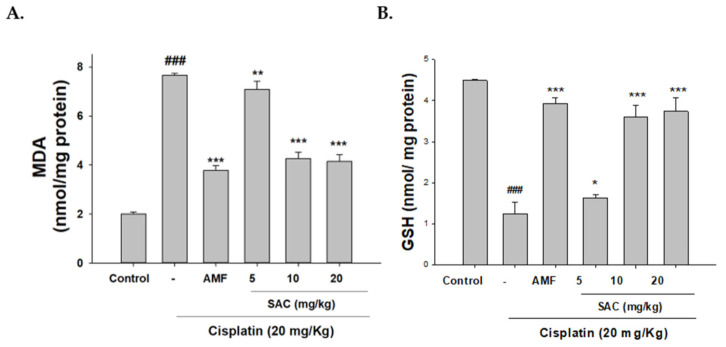
SAC prevents oxidative stress in mice with cisplatin-induced nephrotoxicity. MDA levels (**A**) and GSH levels (**B**) determined by MDA and GSH assays after we homogenized the kidney tissue. The values are reported as the mean ± SEM (*n* = 6). ^###^ *p* < 0.001 compared with the control group. * *p* < 0.05, ** *p* < 0.01 and *** *p* < 0.001 compared with the cisplatin-only group.

Acute kidney disease is linked to oxidative stress. We next determined whether SAC treatment altered oxidative stress in our mouse model. As shown in Figure 3A,B cisplatin significantly reduced the antioxidant capacity of glutathione (GSH) levels and increased MDA levels. Additionally, SAC treatment significantly reduced MDA levels and improved the GSH content. Therefore, SAC pretreatment can reduce oxidative stress in cisplatin-induced nephrotoxic mice.

### 3.5. SAC Attenuated Cisplatin-Induced Inflammation in Renal Tissues

**Figure 4:**
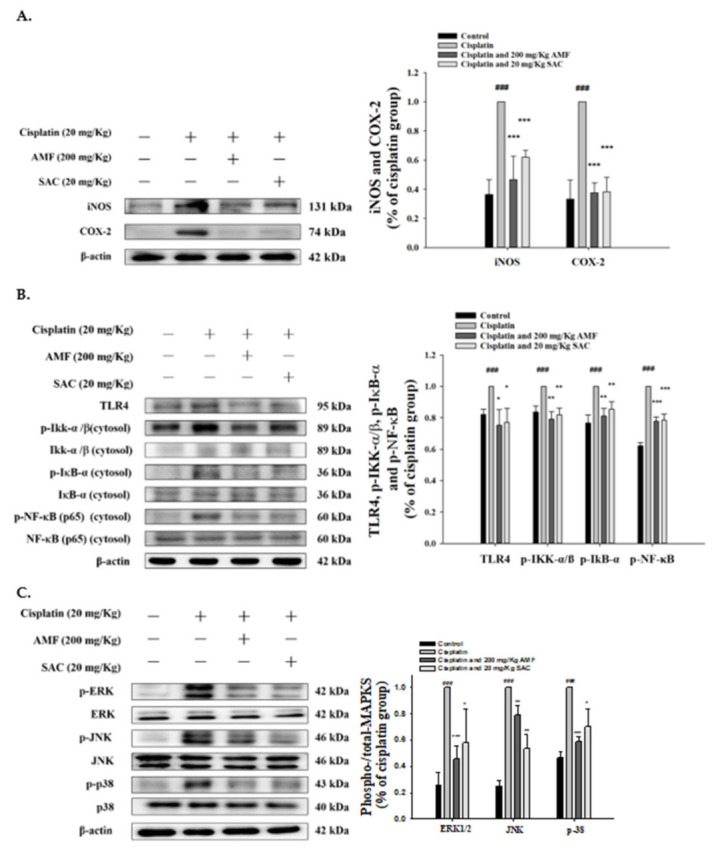
SAC inhibited the cisplatin-induced (**A**) iNOS, COX-2, (**B**) TLR-4, IκB-α and NF-κB, and (**C**) MAPK phosphorylation protein levels in mice with cisplatin-induced nephrotoxicity. Protein levels of iNOS, COX-2, TLR-4, IκB-α, NF-κB and MAPK phosphorylation protein expression in renal tissues were analyzed by Western blot after cisplatin induction. Protein bands were analyzed by densitometric analysis. The values are reported as the mean ± SEM (*n* = 3). ^###^ *p* < 0.001 compared with the control group. * *p* < 0.05, ** *p* < 0.01 and *** *p* < 0.001 compared with the cisplatin-only group.

As shown in Figure 4A, the expression levels of iNOS and COX-2 proteins significantly improved in the cisplatin group and the levels of iNOS and COX-2 proteins reduced in the renal tissues of the SAC group compared to that of cisplatin-induced group.

Toll-like receptor (TLR) is an immunosensor recognizing a variety of endogenous and exogenous molecules in AKI and triggers the intracellular signaling pathways related to renal damage. TLR-4 plays a key role in the pathophysiology of AKI and can be a promising therapeutic target to alleviate kidney damage because of these pathological stimuli. Figure 4B shows cisplatin-induced TLR-4 activation in renal tissues determined by Western blotting. In contrast, SAC pretreatment significantly inhibited the increase in TLR-4 in AKI mice. Thus, the SAC-regulated TLR-4 axis mediates cisplatin-induced production.

The activation of the NF-κB axis is critical for the generation of inflammatory pathways and is related to various human diseases, including kidney diseases [11,12]. Cisplatin-induced NF-κB, IκB kinase (IKK) and IκBα activation in renal tissues are indicated by increased p-NF-κB, p-Ikk and p-IκBα, detected by Western blotting (Figure 4B). Conversely, SAC pretreatment significantly decreased the level of renal p-NF-κB, p-Ikk and p-IκBα in the kidneys of AKI mice, thereby regulating the NF-κB signaling pathway in cisplatin-associated nephrotoxicity. 

### 3.6. SAC-Inactivated Cisplatin Induces the MAPK Pathway in Kidneys

MAPKs play a central role in regulating cisplatin-induced renal damage and inflammation [29,30]. As shown in Figure 4C, p-JNK, p-ERK and p-p38 (the key components of the MAPK signaling pathway) in the kidneys significantly increased by cisplatin but were attenuated by SAC and AMF. The changes in MAPK expression were detected only at phosphorylation levels but not at total protein levels. The data show that SAC inactivated the level of phosphorylated MAPK proteins in cisplatin-induced kidney injury. 

### 3.7. SAC Restores Renal Antioxidant Defense and the HO-1/Nrf2 Signaling Pathway in Cisplatin-Associated Nephrotoxicity

**Figure 5:**
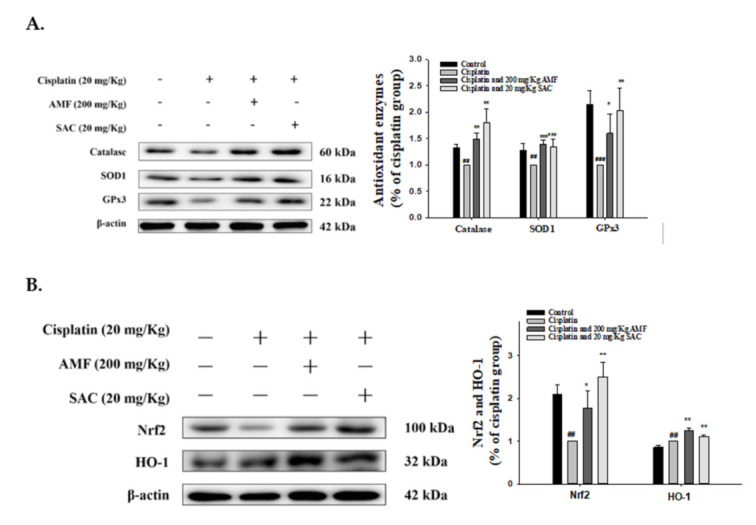
In renal tissues, effects of SAC on the protein expression induced by cisplatin, including (**A**) anti-oxidative enzymes (catalase, SOD1 and GPx3) and (**B**) HO-1 and Nrf2. The protein levels of anti-oxidative enzymes HO-1 and Nrf2 protein expression in renal homogenates were assessed by Western blot analysis after a cisplatin challenge. The analysis of protein bands was carried out by densitometric analysis. The values are reported as the mean ± SEM (*n* = 3). ^##^ *p* < 0.01 and ^###^ *p* < 0.001 compared with the control group; * *p* < 0.05, ** *p* < 0.01 and *** *p* < 0.001 compared with the cisplatin-only group.

Oxidative stress should be regarded as the major cause of renal damage [31]. As shown in Figure 5A, cisplatin induction inhibited the kidney antioxidant defense, as observed by a decrease in the levels of protein expressions of catalase, SOD1 and GPx3, but pretreatment with SAC for catalase, SOD1 and GPx3 recovered these conditions to a near-normal range. Furthermore, the cisplatin group demonstrated a decrease in Nrf2 expression and an increase in HO-1 expression compared with the control group (Figure 5B). In addition, treatment with SAC upregulated the expression of Nrf2 and HO-1 when compared with the cisplatin group (Figure 5B). Taken together, these findings suggest that SAC would be able to improve the related anti-oxidative enzyme protein expression after a cisplatin challenge.

### 3.8. SAC Decreases the Cisplatin-Induced Apoptosis Signaling Pathway

**Figure 6:**
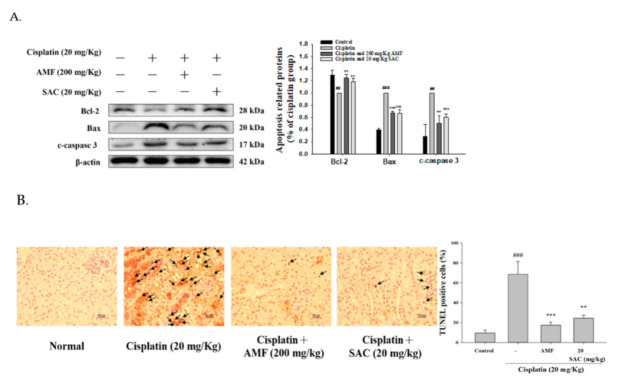
(**A**) SAC reduced the expression of Bcl-2, Bax and cleaved caspase 3 proteins in cisplatin-induced AKI mice. After homogenizing kidney tissue, we determined the protein levels of Bcl-2, Bax and cleaved caspase 3 by Western blot analysis (*n* = 3). (**B**) Histological examination of morphological changes in kidney tissues. Renal tissues stained with TUNEL (400×). Renal tubular cell apoptosis, and the presence of TUNEL-positive cells were measured by an image analyzer (*n* = 6). ^##^ *p* < 0.01 and ^###^ *p* < 0.001 compared with the control group; ** *p* < 0.01 and *** *p* < 0.001 compared with the cisplatin-only group. Arrows show renal tubular cell apoptosis.

Accumulated evidence showed that renal tubular cell apoptosis exacerbated the pathogenesis of cisplatin-induced AKI. As shown in Figure 6A, the cisplatin group upregulated the protein levels of Bax and cleaved caspase 3 and decreased the expression of Bcl-2 compared to the control group. Otherwise, SAC pretreatment significantly suppressed the levels of Bax and cleaved caspase 3 and increased the levels of Bcl-2 in response to a cisplatin challenge.

As shown in Figure 6B, compared to the normal group, more TUNEL-stained cells were observed in the cisplatin group and SAC (20 mg/kg) significantly attenuated tubular cell apoptosis. These data demonstrate that SAC decreased tubular cell apoptosis under cisplatin exposure in our mouse model.

### 3.9. SAC Alleviates the Cisplatin-Induced CaMKK–AMPK–Sirt1 Axis

**Figure 7:**
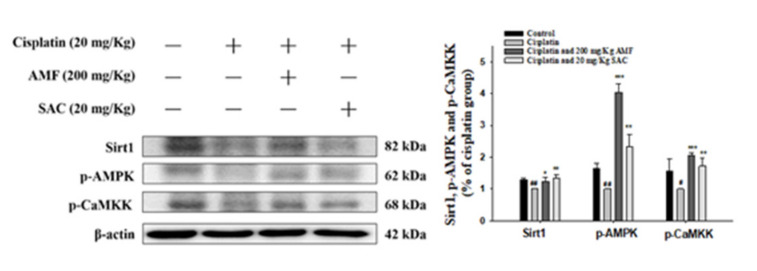
SAC inhibited Sirt1, p-CaMKK and p-AMPK protein expression in cisplatin-induced AKI mice. After homogenizing kidney tissue, we determined the protein levels of Sirt1, p-CaMKK and p-AMPK by Western blot analysis. The values are reported as the mean ± SEM (*n* = 3). ^#^ *p* < 0.05 and ^##^ *p* < 0.01 compared with the control group; * *p* < 0.05, ** *p* < 0.01 and *** *p* < 0.001 compared with the cisplatin-only group.

Under oxidative stress, phosphorylation of CaMKK and AMPK can increase Sirt1 expression to control energy homeostasis and stress response [32]. As shown in Figure 7, cisplatin decreased the amounts of p-CaMKK, p-AMPK and Sirt1 proteins. Furthermore, SAC pretreatment markedly elevated the levels p-CaMKK, p-AMPK and Sirt1 in kidney tissues of AKI mice (Figure 7). These data demonstrate that SAC raised the expression of p-CaMKK, p-AMPK and Sirt1 proteins under cisplatin exposure in our mouse model. 

### 3.10. Blocking Sirt1 Synergy with EX-527 Increases Kidney Failure with Cisplatin-Induced Nephrotoxicity

**Figure 8:**
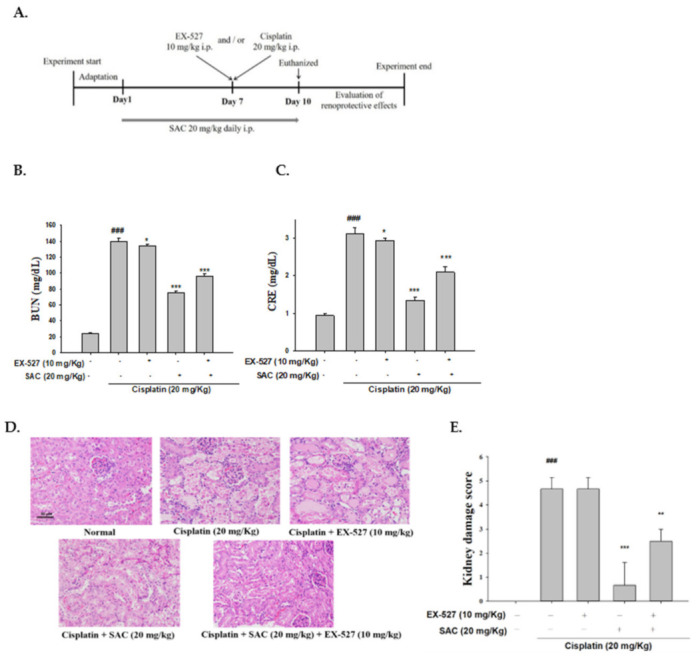
Effects of SAC and the Sirt1 inhibitor (EX-527) on cisplatin-induced nephrotoxicity. SAC and/or EX-527 given to mice at a daily intraperitoneal (i.p.) dose of 20 mg/kg and/or 10 mg/kg for 10 days; they were given cisplatin 20 mg/kg, i.p. 1 h after SAC and/or EX-527 administration on day 7 and were euthanized on day 11. (**A**) Experiment design, (**B**) blood BUN levels, (**C**) serum CRE levels, (**D**) kidneys stained with H&E and (**E**) the kidney injury scores. Each group’s kidneys were provided for histological evaluation. After staining, representative histological sections were magnified (400×) and photographed. The values are reported as the mean ± SEM (*n* = 6). ^###^ *p* < 0.001 compared with the control group. * *p* < 0.05, ** *p* < 0.01 and *** *p* < 0.001 compared with the cisplatin-only group. Arrows show tubular cell necrosis; scale bar = 50 μm.

As shown Table 2, mice treated with cisplatin show significant weight loss and elevated relative kidney indexes compared with normal mice. However, SAC and/or EX-527 pretreatment induces a significantly higher resistance to cisplatin-induced nephrotoxicity, such as reduction in the kidney index and increase in the body weight (Table 2). Moreover, the group pretreated with only EX-527 had significantly higher levels of CRE and BUN under cisplatin exposure (Figure 8B,C). Pretreatment with EX-527 and SAC highly decreased the serum CRE and BUN levels.

Subsequently, the histopathological changes were analyzed to determine whether EX-527 and/or SAC has an effect on kidney failure after a cisplatin challenge. The renal tissue of the control group was regular, and the mice pretreated with only EX-527 showed significantly increased necrosis and inflammatory infiltrating cells under cisplatin exposure. Mice pretreated with EX-527 and SAC showed significantly decreased renal dysfunction compared with the cisplatin-only group (Figure 8D,E).

### 3.11. SAC Demonstrate the Anti-Inflammatory Effect when Administering EX-527 as Sirt1 Blocker

**Figure 9:**
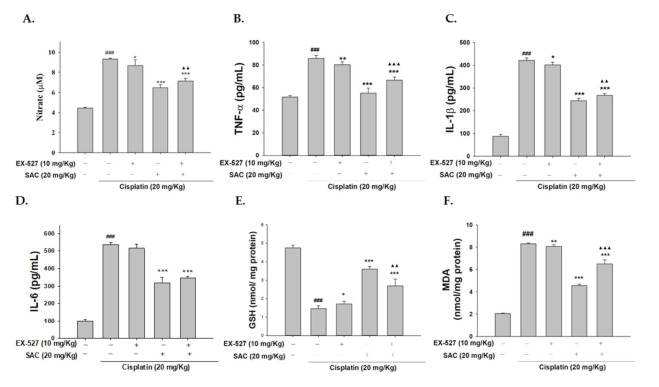
SAC and the Sirt1 inhibitor (EX-527) changed the NO (**A**), TNF-α (**B**), IL-1β (**C**), IL-6 (**D**), GSH (**E**) and MDA (**F**) levels in cisplatin-induced AKI mice. TNF-α, IL-1β and IL-6 levels in the serum of the mice were determined by commercial ELISA kits. The values are reported as the mean ± SEM (*n* = 6). *^###^ p* < 0.001 compared with the control group. * *p* < 0.05, ** *p* < 0.01 and *** *p* < 0.001 compared with the cisplatin-only group. ^▲▲^ *p* < 0.01 and ^▲▲▲^ *p* < 0.001 compared with the SAC + cisplatin group and the EX-527 + SAC + cisplatin group.

To determine that the Sirt1 inhibitor (EX-527) can inhibit the CaMKK–AMPK–Sirt1 signaling pathway, we evaluated the expression of the proteins related to the CaMKK–AMPK–Sirt1 axis in the cisplatin + EX-527 group and the cisplatin group. Both groups showed significantly increased expression of pro-inflammatory cytokines (NO, TNF-α, IL-1β and IL-6) and MDA and decreased expression of GSH. Furthermore, the SAC + EX-527 group showed a significantly decreased expression of pro-inflammatory cytokines and MDA and increased the expression of GSH compared to the cisplatin group (Figure 9A–F). SAC partially inhibited cisplatin toxicity through Sirt1. In addition, the group pretreated with SAC + cisplatin showed significantly decreased NO, TNF-α, IL-1β and MDA levels and a increased GSH level compared to the EX-527 + SAC + cisplatin group. Collectively, the data indicate that SAC suppresses the activity of the CaMKK–AMPK–Sirt1 pathways in cisplatin-induced AKI mice. 

## 4. Discussion

Cisplatin, a platinum compound, is currently applied singly or in combination with other medicines to handle different types of cancers, including bladder, head and neck, lung, ovarian and testicular cancers [1]. The mortality rate of AKI is as high as 50% and surviving AKI patients have a high chance of developing chronic kidney disease within months to years [2]. Regrettably, there is currently no beneficial therapy to avoid AKI caused by cisplatin [3,33]. There is an urgent need to develop new treatment options for this severe disease. 

*S. miltiorrhiza*, an important herb in traditional Chinese medicine, has been extensively applied for thousands of years in China to treat cardiovascular and cerebrovascular disease. Salvianolic acids as the most abundant water-soluble component extracted from *S. miltiorrhiza* have attracted increasing attention from scientists due to their comprehensive anticancer actions [34]. Salvianolic acids have emerged as potent anti-cancer molecules. They fight cancer progression by prompting apoptosis, halting cell cycle and adjourning metastasis by targeting multiple deregulated signaling networks of cancer [35]. Previous research has indicated that salvianolic acid A reverses cisplatin resistance by targeting c-met and attenuating the Akt–mTOR pathway in lung cancer [36] and salvianolic-acid-B-attenuated cisplatin-induced cardiac injury and oxidative stress by modulating the Nrf2 signal pathway. In addition, the molecular mechanism of cisplatin in the treatment of kidney injury remains unclear. However, recent evidence indicates that oxidative stress and inflammation take the lead in cisplatin-induced nephrotoxicity [37]. Here, intraperitoneal administration of cisplatin was used to induce AKI in mice as a new animal model [38]. We further applied this mouse model to examine the defensive effect of SAC against oxidative and inflammatory stresses, the purpose of the research being to examine the protective effects of SAC on cisplatin-challenged kidney injury in vivo. We used non-cytotoxicity and the highest dose of SAC (20 mg/kg) to perform a Western blot experiment to explore its pathway. Then, we used AMF (positive control), a prodrug, which is phosphorylated by the alkaline phosphatase enzyme to its active metabolite. In the preclinical studies, AMF selectively protects normal cells against the lethal effects of chemotherapy and radiotherapy and increases the efficacy of the treatment by decreasing the dose-limiting toxic effects. Experimental and clinical trials have shown that AMF does not alter the antitumor activity of chemotherapy or radiotherapy. AMF is currently recommended for the prevention of cisplatin-induced nephrotoxicity [39,40,41]. In this study, we used AMF and SAC to treat cisplatin-induced AKI were effective in vivo. Since the research and development of drugs based on natural products is very popular in recent years, because of fewer side effects and high bioactive potential. Thus, the active compounds from the herbal plants exert a broad range of pharmacological activities and have been used to make herbal medicine and some modern drugs. Therefore, the pharmacological effects of SAC have been found by a large number of studies that SAC has antioxidant, anti-cancer, anti-inflammatory and antioxidant properties, can protect various organs from diseases and can be used as an auxiliary food for chemotherapy or as a prodrug for the development of new drugs.

Nephrotoxicity is a major challenge in the application of cisplatin as a potent drug to cure various cancers. After being injected, cisplatin accumulates in renal tubular cells, causing renal insufficiency and increasing the levels of CRE and BUN. Treatment with SAC decreased the levels of BUN and CRE on day 10 in mice with cisplatin-induced AKI, suggesting that SAC enhances kidney function. These findings further support that SAC can serve as a potential protective agent for cisplatin-challenged AKI. 

Cisplatin induction has been shown to cause severe kidney damage and histological changes, such as tubular necrosis and tubular cell damage, including vacuolar degeneration and detachment [42]. In this study, dilation of renal tubules and severe tubular necrosis were also observed in the cisplatin-treated mice. Due to the protective effects of SAC, administration of cisplatin caused little histological changes in the treatment group, and the group administered SAC showed almost no dilated tubules and tubular necrosis, proving the protective effects of SAC against cisplatin-associated kidney toxicity.

Inflammation plays a key in the pathogenesis of cisplatin-associated AKI [40]. Cisplatin can directly accumulate in proximal tubules and cause renal cytotoxicity and pro-inflammatory cytokines also aggravate kidney damage. The present study demonstrated that SAC decreases the levels of pro-inflammatory cytokines in the kidney after a cisplatin challenge and blocks the cytokine-associated signaling axis, which could be important in conceptualizing mechanisms for SAC to ameliorate cisplatin-associated AKI [9,10].

NF-kB plays a key role in inflammation by regulating genes encoding pro-inflammatory factors. Cisplatin-induced stress factors activate the phosphorylation of IκB protein, subsequently degrading and releasing NF-κB. Activated NF-κB translocates into the nucleus and induces the production of COX-2, iNOS and pro-inflammatory cytokines in renal tubular cells [43,44]. To investigate the anti-inflammatory action of cisplatin-treated stress, its effect was tested on NF-κB activation by using phosphate-NF-κBp65 (p-p65). In a study, the expression levels of COX-2, iNOS, p-IκB and p-NF-κB proteins were considerably increased after cisplatin exposure. However, after SAC treatment, the expression of these proteins was inhibited. These data suggest that SAC can prevent cisplatin-induced renal toxicity by inhibiting the inflammatory pathway.

TLR-4 is a receptor protein that mediates the NF-κB axis and the pro-inflammatory signaling functions [12]. TLR-4 activation could lead to inflammation and subsequent kidney injury [12]. TLR4 expression in murine peritoneal macrophage was increased by cisplatin treatment in vitro [45]. TLR4 was essential to the initiation of intrarenal inflammatory mediator production in cisplatin-induced nephrotoxicity [46]. Cisplatin synergistically acted with TLR4-specific ligand, lipopolysaccharides (LPS), to produce inflammatory cytokines such as TNF-α and IL-6, thereby leading to nephrotoxicity [47]. In TLR4-deleted mice, cisplatin-induced inflammation and renal injury were significantly reduced compared with wild-type mice [46]. Our results demonstrated that an increase in TLR-4 and p-NF-κB levels after cisplatin treatment could be inhibited by SAC. Other studies have shown that the activation of NF-κB is related to cisplatin-induced nephrotoxicity in patients and animal models [48]. After cisplatin induction, the protein expression of TLR-4 showed increase and further studies are needed to prove their correlation.

MAPKs play an important role in regulating cisplatin-induced kidney damage and inflammation [29,30]. In the present study, we provided evidence that the phosphorylation of MAPKs was significantly inhibited, revealing that SAC reduces the onset of acute renal failure after cisplatin administration. SAC also prevents an increase in the levels of pro-inflammatory cytokines by activating NF-κB in cisplatin-induced models. Taken together, SAC could significantly prevent the degradation of IκB-α and the phosphorylation of NF-κB and MAPK after cisplatin exposure.

Oxidative stress is one of the most important elements in cisplatin-associated acute renal failure, followed by the accumulation of ROS [49,50]. Recent evidence suggests that oxidative stress can change histopathology by increasing the formation of lipid peroxidation products and reducing antioxidant enzyme expression [51]. These data suggest that SAC improves the expression of antioxidant proteins and the amount of GSH and decreases the formation of MDA after a cisplatin challenge. In addition, oxidative stress triggers the release of Nrf2 from the Nrf2–Keap2 complex, thereby inducing the expression of Nrf2 and its related genes downstream, which play a vital role in the ability to inhibit inflammation through antioxidant pathways. Nrf2 is a transcription factor; therefore, it increases the expression of genes encoding antioxidant-related proteins, such as HO-1, GPx and GSH-S-transferase, which protect tissues by removing the oxidative damage caused by free radicals [52]. Our experimental results revealed that the defensive effect of SAC regulates the Nrf2–HO-1 axis after cisplatin treatment. However, SAC pretreatment can effectively reverse oxidative stress changes to ensure relative normal renal function.

Numerous studies have shown that the Bcl-2 family and the caspase family play a critical regulatory role in the apoptotic pathway. Cisplatin causes DNA renal damage through translocation of Bcl-2 family proteins, which are associated with caspase-3-dependent apoptosis [38]. Apoptosis-associated proteins of various substrates can trigger the apoptotic responses that cause cisplatin nephrotoxicity [40]. In the present study, our results showed that SAC treatment significantly decreased the cisplatin-induced cellular apoptosis by down-regulating the expressions of Bax and cleaved-caspase 3 protein and increasing the expression of Bcl-2 protein. These results imply that SAC could alleviate cisplatin-induced AKI by regulating the apoptosis pathway.

The CaMKK–AMPK–Sirt1 axis is associated with the adjustment of inflammatory cytokines release [53,54]. CaMKK is an essential signaling molecule that is activated to increase intracellular calcium and is responsible for regulating inflammation and immunity [55]. Recently, for muscle cells and macrophages, the phosphorylation of CaMKK has been reported as possibly caused by calcium influx or LPS induction [56]. AMPK is a central sensor of energy balance, and activation of AMPK leads to significant anti-inflammatory and immunosuppressive effects. AMPK has recently been identified as a direct substrate of CaMKK (being able to regulate the inflammation of macrophages), and a currently recognized molecular model can explain the anti-inflammatory effects of CaMKK [53,54]. In addition, the molecular mechanism of AMPK is more complex, as AMPK can suppress NF-κB activation and inhibit the inflammatory response as well as regulate its downstream target molecules, including Sirt1, peroxisome-proliferator-activated receptor γ coactivator-1α (PLC-1α), p53 and Forkhead box class O 3a (FOXO3a) [57]. Recently, AMPK synergistically achieved a key regulatory incident of Sirt1 regulatory expression by improving the NAD/NADH ratio. Sirt1 regulates cell metabolism and reduces ROS-induced apoptosis, bringing about a long life and resistance to oxidative stress. Thus, AMPK may act as an important regulator of Sirt1 expression because of the processes involved in catabolism, activation of mitochondria and cell survival [58,59]. Taken together, our data indicate cisplatin-associated AKI via the decreased expression of Sirt1, p-AMPK and p-CaMKK, and their expression might increase after treatment with SAC.

Several inflammation-related proteins have been found to mediate Sirt1, such as NF-κB [60]. Thus, the data indicate that the specific inhibitor of Sirt1, EX-527, increased the activation of inflammatory pathways in mice, while SAC pretreatment in mice effectively reversed these inflammatory response changes compared to the cisplatin-treated group in mice.

## 5. Conclusions

**Figure 10:**
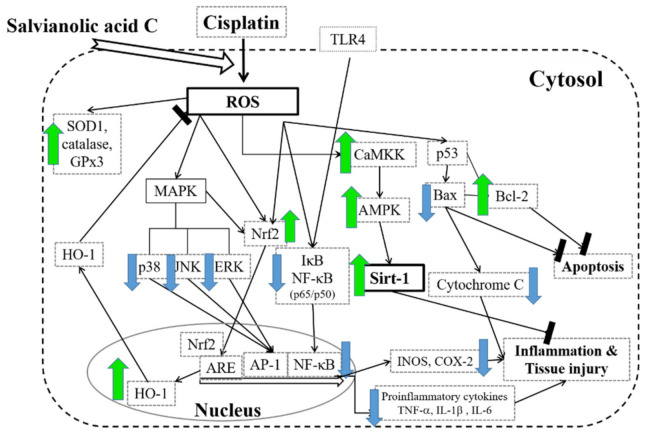
A scheme displaying the protective effect of SAC against cisplatin-associated renal injury. The green arrows indicate an increase. The blue arrows indicate a decrease. ROS: reactive oxygen species; MAPK: mitogen-activated protein kinase; JNK: C-jun NH2-terminal kinase; ERK: extracellular-signal-regulated kinase; ARE: antioxidant response element; AP-1: activator protein 1; NF-κB: nuclear factor of κB; HO-1: heme oxygenase 1; SOD1: Cu/Zn superoxide dismutase; GPx3: glutathione peroxidases 3; Nrf2: nuclear-factor-erythroid-2-related factor 2; TLR-4: toll-like receptor 4; IκB: inhibitor of the nuclear factor kappa B; CaMKK: calcium/calmodulin-dependent protein kinase kinase; AMPK: 5’-adenosine-monophosphate-activated protein kinase; Sirt1: Sirtuin-1; iNOS: inducible nitric oxide synthase; COX-2: cyclooxygenase-2; p53: tumor protein p53; Bax: Bcl-2-associated X; Bcl-2: B-cell lymphoma 2; TNF-α: tumor necrosis factor-α; IL-1ß: interleukin-1β; IL-6: interleukin-6.

In this article, we showed for the first time that SAC, one of the major polyphenolic compounds of *Salvia miltiorrhiza*, controls the inflammatory effects in the animal model of AKI induced by cisplatin by the suppression if kidney histopathologic changes, infiltration of inflammatory cell and the release of pro-inflammatory cytokines. SAC can be developed as a promising therapeutic agent to provide potent anti-inflammatory and antioxidant effects against cisplatin-associated AKI, mediated by inhibiting the TLR-4-NF-κB–MAPK-, HO-1–Nrf2- and CaMKK–AMPK–Sirt1-associated signaling axes (Figure 10). Previous studies have shown the renoprotective effect of SAC only in a mouse model of cisplatin nephrotoxicity. Obviously, more studies involving clinical trials on humans with AKI are needed. In conclusion, SAC inhibits inflammation and ROS in cisplatin-associated AKI to prevent kidney injury.

## Figures and Tables

**Table 1:** SAC changes the body weight and the kidney index in cisplatin-associated nephrotoxicity. The data are presented as the mean ± SEM (*n* = 6). ^###^ *p* < 0.001 compared with the control group. ** *p* < 0.01 and *** *p* < 0.001 compared with the cisplatin-only group.

Groups	Initial Body Weight (g)	Final Body Weight (g)	Kidney Index (mg/g)
Control	29.8 ± 0.53	31.87 ± 0.45	1.34 ± 0.02
Cisplatin (20 mg/kg)	29.93 ± 0.65	25.72 ± 0.98 ^###^	2.22 ± 0.08 ^###^
Cisplatin (20 mg/kg) + AMF (200 mg/kg)	30.03 ± 0.21	30.27 ± 0.21 ***	1.51 ± 0.02 ***
Cisplatin (20 mg/kg) + SAC (5 mg/kg)	29.85 ± 0.39	26.62 ± 0.52	2.07 ± 0.07 **
Cisplatin (20 mg/kg) + SAC (10 mg/kg)	30.1 ± 0.28	29.1 ± 0.53 ***	1.66 ± 0.04 ***
Cisplatin (20 mg/kg) + SAC (20 mg/kg)	30.0 ± 0.37	29.45 ± 0.17 ***	1.56 ± 0.02 ***

**Table 2:** SAC and the Sirt1 inhibitor (EX-527) change the body weight and the kidney index, showing resistance to cisplatin-associated nephrotoxicity. The values are reported as the mean ± SEM (*n* = 6). ^###^ *p* < 0.001 compared with the control group. ** *p* < 0.01 and *** *p* < 0.001 compared with the cisplatin-only group.

Groups	Initial Body (g)	Final Body (g)	Kidney Index (mg/g)
Control	33.78 ± 0.66	37.39 ± 1.08	1.44 ± 0.04
Cisplatin (20 mg/kg)	33.39 ± 0.30	32.41 ± 0.62 ^###^	2.46 ± 0.04 ^###^
Cisplatin (20 mg/kg) + SAC (20 mg/kg)	33.65 ± 0.30	34.96 ± 0.51 ***	1.55 ± 0.02 ***
Cisplatin (20 mg/kg) + EX-527 (10 mg/kg)	33.43 ± 0.33	32.86 ± 0.22	2.39 ± 0.04 **
Cisplatin (20 mg/kg) + SAC (20 mg/kg) + EX-527 (10 mg/kg)	33.46 ± 0.19	33.9 ± 0.58 **	1.69 ± 0.03 ***

## Data Availability

The data presented in this study are available on request from the corresponding author.
